# Hypothalamic astrocytes: connecting brain and periphery in metabolic control

**DOI:** 10.1007/s11154-025-09995-0

**Published:** 2025-10-13

**Authors:** Ophélia Le Thuc, Franziska M. Lechner, Cristina García-Cáceres

**Affiliations:** 1 Institute for Diabetes and Obesity, Helmholtz Diabetes Center at Helmholtz Zentrum München, Neuherberg, 85764 Germany; 2 German Center for Diabetes Research (DZD), Neuherberg, 85764 Germany; 3 Medizinische Klinik Und Poliklinik IV, Klinikum Der Universität, Ludwig-Maximilians-Universität München, Munich, 80336 Germany

**Keywords:** Astrocytes, Hypothalamus, Sympathetic nervous system, Sexual dimorphism, Obesity

## Abstract

Astrocytes, once viewed primarily as supportive cells in the central nervous system, are increasingly recognized as dynamic regulators in the regulation of systemic metabolism, especially within the hypothalamus. Recent research highlights their capacity to sense and integrate nutrient, hormonal, and circadian signals, modulate hypothalamic circuits, and ultimately influence whole-body energy balance. In this review, we discuss emerging studies that explore how hypothalamic astrocytes regulate glucose and lipid metabolism through neuroendocrine and autonomic pathways that extend their influence to peripheral organs. We examine emerging evidence showing that astrocytes contribute to glycemic control via glucose sensing, intracellular metabolic shifts, and modulation of key neuronal circuits. Similarly, recent investigations have identified roles for astrocytes in the regulation of adipose tissue function and body weight, particularly in the context of high-fat diet exposure, largely through their influence on hypothalamic neuron-astrocyte interactions and sympathetic output. We further consider recent findings implicating astrocytic circadian pathways in the coordination of metabolic rhythms, as well as the long-term consequences of early-life nutritional exposures, that may epigenetically program hypothalamic astrocyte function. New insights also point to region-specific and sex-dependent astrocytic functions. Together, this growing body of work positions hypothalamic astrocytes as integrators of brain-body communication in the control of energy homeostasis and highlights their potential relevance in the pathophysiology of obesity and metabolic disease.

## Hypothalamic astrocytes as specialized regulators of central energy homeostasis

Astrocytes, long overlooked in comparison to neurons in the study of central metabolic regulation, are now recognized as integral and dynamic contributors to the central regulation of systemic energy balance. They are increasingly recognized not merely as passive support cells, but as active participants in central nervous system function. This is particularly evident in the hypothalamus, where astrocytes are strategically positioned between neurons, blood vessels, and the cerebrospinal fluid, placing them at the interface of multiple metabolic and hormonal signals. This interface is most prominent in the arcuate nucleus, a region adjacent to the third ventricle (3 V) and the median eminence (ME), where the blood–brain barrier (BBB) exhibits increased permeability to circulating signals. These anatomical features enable astrocytes to rapidly detect and respond to fluctuations in systemic nutrient and hormone levels, positioning them as key metabolic sensors within the hypothalamus.

Astrocytes are specialized glial cells and functional components of synapses, and they are intimately associated with vascular networks. These cells regulate the properties of the BBB and cerebral blood flow via their endfeet, and facilitate the trafficking of signals between the bloodstream and the brain. Through the release of soluble active factors, they modulate the extracellular matrix, promote the remodeling of local vasculature, and regulate the exchange of nutrients, hormones, and metabolites, processes that are essential for coordinated brain-periphery communication [1, 2].

In the hypothalamus, astrocytes are recognized as central players in nutrient and hormone sensing. They regulate glucose uptake into the brain via glucose transporter 1 (GLUT1), of which the expression and localization change in response to the metabolic status [3, 4]. Astrocytic insulin receptor (IR) signaling directly influences GLUT1 expression and glycolytic activity. Conditional deletion of IRs in astrocytes reduces glucose uptake, decreases lactate efflux, and triggers compensatory increases in fatty acid β-oxidation, thereby impairing energy substrate supply to neurons [5, 6]. This disruption also affects the interactions between astrocytes and pro-opiomelanocortin (POMC) neurons, reducing the glucose sensitivity of POMC neurons, a key anorexigenic population involved in energy homeostasis. The active role of astrocytes in central glucose sensing is further supported by findings that activation of astrocytes in the paraventricular nucleus of the hypothalamus (PVN) leads to a bidirectional modulation of the activity of surrounding neurons, but also of autonomic outflow, to ultimately regulate systemic glucose sensing and energy balance [7].

Astrocytes also express leptin receptors (LepR) and exhibit morphological and molecular changes in response to leptin, including increased expression of the marker glial fibrillary acidic protein (GFAP) [8]. Astrocytic LepR signaling is necessary for the proper structural and functional organization of hypothalamic feeding circuits. Conditional LepR deletion in astrocytes alters astrocyte-neuron interaction, reduces leptin-induced STAT3 phosphorylation, and impairs leptin-induced suppression of food intake [9, 10]. These mice exhibit enhanced feeding responses to ghrelin and fasting, indicating disrupted integration of hormonal signals [9, 10]. Interestingly, astrocytes also display some structural and molecular changes in response to fasting or ghrelin [11, 12].

Astrocytes also participate in lipid sensing and metabolism. They express lipoprotein lipase (LPL), and astrocyte-specific LPL deletion leads to the accumulation of ceramides in the melanocortin neurons, a lipid species implicated in neuronal dysfunction and lipotoxicity [13, 14]. Additionally, astrocytes produce endozepines, such as octadecaneuropeptide (ODN), derived from acyl-CoA binding protein (ACBP). Deletion of astrocytic ACBP promotes hyperphagia and weight gain, while its overexpression suppresses food intake and enhances POMC neuron activity, supporting an anorexigenic role to this gliopeptide via POMC neurons [15]. More recent work suggests a context-dependent role for astroglial ACBP: its deletion does not impair weight loss in obese mice switched from a high-fat diet (HFD) to chow, nor alter metabolic parameters under fasting, thermoneutrality, or cold stress, but significantly disrupts feeding behavior during refeeding and cold exposure [16]. Rather than acting solely as an anorexigenic factor, ACBP may contribute to bidirectional modulation of energy intake, depending on physiological context and metabolic state.

Functionally, astrocytes modulate synaptic transmission and plasticity via Ca^2+^-dependent gliotransmitter release [17]. Activation of astrocytes in the mediobasal hypothalamus (MBH) suppresses food intake through adenosine A1 receptor-mediated inhibition of Agouti-related protein (AgRP)-expressing neurons [18]. Conversely, optogenetic stimulation of arcuate astrocytes increases food intake via AgRP neuron activation [19], suggesting region- and context-dependent regulation of hunger-promoting AgRP neurons by astrocytes.

Diet-induced obesity (DIO) models have revealed early and robust astrocytic responses to hypercaloric diets. Hypothalamic astrocytes exhibit rapid and transient upregulation of GFAP and aldehyde dehydrogenase 1 family member L1 (Aldh1L1), occurring even before significant weight gain or systemic inflammation [20–22]. These changes are accompanied by morphological remodeling and altered synaptic coverage, especially in the arcuate nucleus, which is in direct contact with fenestrated capillaries of the ME and third ventricle [3, 8]. Notably, this glial reactivity appears transient and reversible upon return to a normocaloric diet [23], though sex-specific and region-dependent variations have been reported [24, 25].

Mechanistically, pro-inflammatory signaling pathways such as IKKβ/NF-κB and Ca^2+^/calcineurin have been implicated in astrocytic activation and metabolic dysfunction. Inhibition of IKKβ in astrocytes reduces gliosis, restores leptin and insulin sensitivity, and improves metabolic parameters in DIO mice [11, 26]. Conversely, chronic activation of this pathway leads to shorter astrocytic process, altered GABA and BDNF regulation, and exacerbated metabolic dysfunction. Calcineurin signaling similarly contributes to the gliotic response in DIO [27].

In metabolic disease, astrocytes, through maladaptive reactivity and a loss of homeostatic support, may also contribute to neurodegenerative-like pathologies. Astrocyte reactivity, which can be induced by microglial cytokines during inflammation, may cause astrocytes to lose their normal supportive functions and secrete neurotoxic factors. Such a phenomenon has been observed in neurodegenerative conditions and could contribute to neuronal death [28]. Similar reactive profiles and inflammatory responses have been observed in the hypothalamic inflammation associated with obesity.

Overall, hypothalamic astrocytes appear metabolically sensitive, transcriptionally dynamic, and regionally specialized regulators of central energy homeostasis. By integrating peripheral metabolic signals and modulating local circuit properties, they act as essential gatekeepers between body and brain in the regulation of systemic metabolism.

Recent advancements in metabolic neuroscience, particularly new methods that enable the study of glial function in freely moving animals while preserving anatomical context, have paved the way for a better understanding of how hypothalamic astrocytes play a critical role at the interface between brain and periphery, thereby regulating key aspects of metabolic control. In this review, we discuss the recent literature to further delineate the emerging roles of hypothalamic astrocytes as key regulators of metabolic control. We emphasize how these cells, through dynamic interactions with their local microenvironment, regulate the transport and signaling of peripheral cues, thereby contributing to the regulation of whole-body energy homeostasis.

## Hypothalamic astrocytes: central regulators of systemic glucose metabolism

While most research on glucose homeostasis has focused on peripheral organs, accumulating evidence underscores the critical role of the brain, particularly the hypothalamus, in coordinating counterregulatory responses to hypoglycemia. Acting as a central glucose-sensing hub, the hypothalamus detects declines in circulating glucose levels and triggers coordinated neuroendocrine and autonomic outputs to restore peripheral glucose availability [29–33]. Emerging findings have further expanded this framework by identifying hypothalamic astrocytes as active participants in glucose sensing. These astrocytes are capable of detecting fluctuations in blood glucose and relaying metabolic information to neighboring glucose-sensitive neurons, thereby shaping the activation of neural circuits that drive the secretion of counterregulatory hormones and promote the restoration of normoglycemia [4, 34]. Interestingly, interfering with lipid metabolism [35, 36] and inflammatory signaling [11, 37] in astrocytes also impairs glucose tolerance. POMC neurons not only have a well-established role in energy balance but are also involved in the response to hypoglycemia to initiate counterregulatory responses, underscoring their dual function in metabolic control [38, 39]. As previously mentioned, increasing evidence suggests that astrocytes actively modulate POMC neuron activity through both morphological and signaling mechanisms. One such regulatory process is the dynamic modulation of glial ensheathment of neurons, which appears sensitive to the organism’s metabolic state. For instance, in postprandial conditions or after glucose administration, glial processes have been shown to retract from POMC neurons, thereby facilitating their activation [40]. Supporting this, a reduction in astrocytic coverage of arcuate POMC neurons was reported following glucose injection in control mice, an effect that was absent in astrocyte-specific IR knockout mice. In these IR-deficient animals, baseline glial coverage was already reduced, suggesting that intact insulin signaling is required for proper astrocytic structural plasticity in response to glucose [5]. These diverse findings reflect not only the functional complexity of astrocytic intracellular signaling but also the anatomical and functional heterogeneity of the hypothalamus itself. The hypothalamus comprises multiple distinct nuclei, each with specialized roles in glucose regulation. Consequently, the metabolic outcomes of manipulating astrocytic functions may vary depending on the specific hypothalamic region and astrocyte-neuron circuits targeted. Some nuclei may drive glucose-lowering effects, while others could promote counterregulatory responses, highlighting the need to consider this regional specificity when interpreting experimental results and evaluating the role of astrocytes in hypothalamic control of systemic glucose metabolism.

**Fig. 1:**
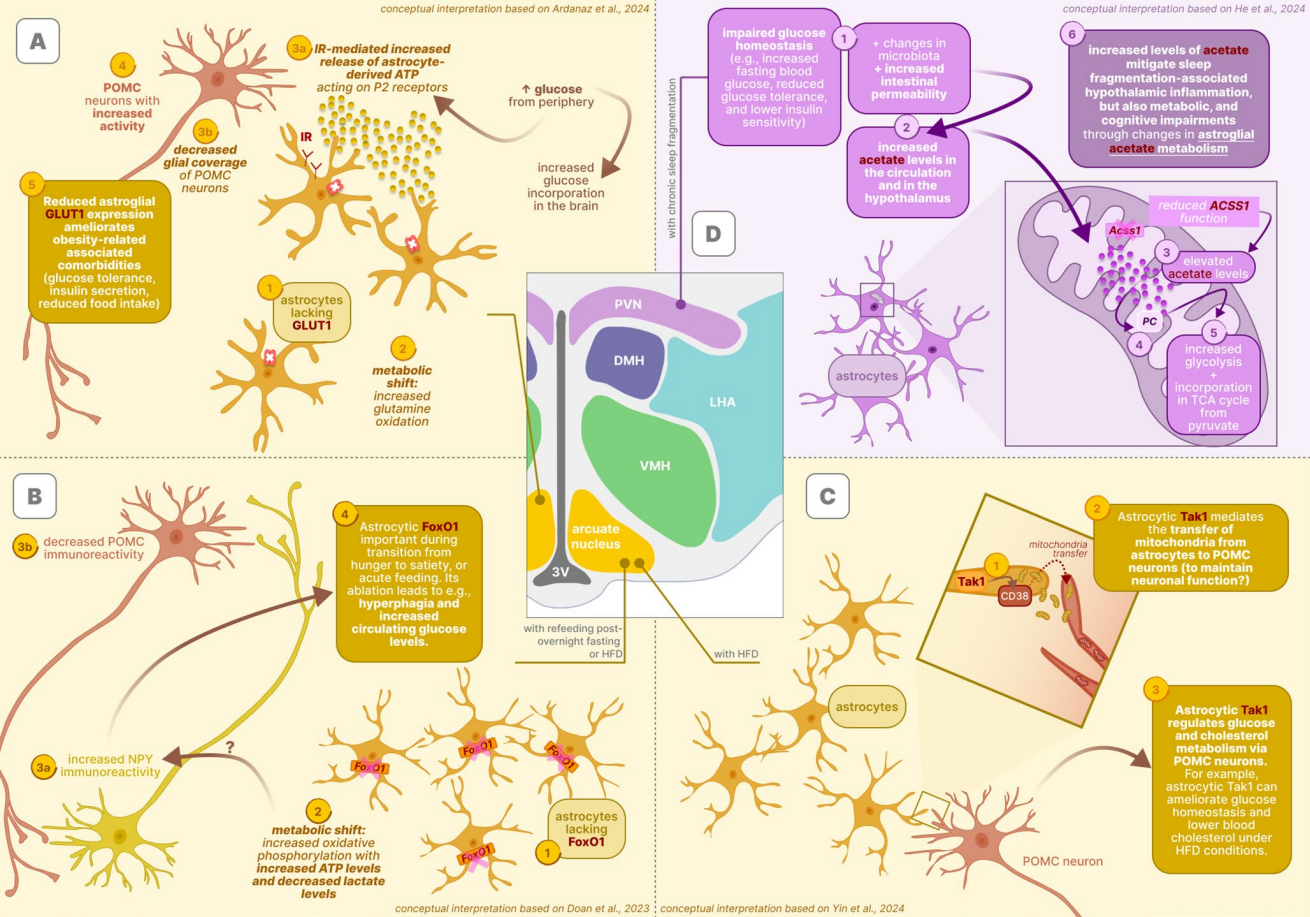
New findings highlight the role of hypothalamic astrocytes in regulating systemic glucose homeostasis. **A** Top left panel (from Ardanaz et al*.*, 2024 (reference 42)): Astrocytic GLUT1 plays a critical role in regulating glucose uptake into the brain, and mediating local central responses to peripheral glucose challenges. The lack of astrocytic GLUT1 (1) led to a compensatory metabolic shift in these glial cells, relying more on glutamine oxidation (2) although without increased ATP production. Nevertheless, the lack of astrocytic GLUT1 ameliorated some metabolic disturbances associated with obesity. The authors have reported that this is associated with an astroglial IR-mediated increase in the secretion of astrocyte-derived ATP, e.g., in response to glucose, which acts on purinergic receptors in the brain (3a), and with a decrease in glial coverage of POMC neurons (3b) of which the activity was found increased (4). **B** Bottom left panel (from Doan et al*.,* 2024 (reference 49)): The deletion of the transcription factor FoxO1 in astrocytes (1) also led to a metabolic shift. Here, astrocytes rely further on oxidative phosphorylation (2). ATP production increased while extracellular lactate levels decreased (2). This was associated with an increase in NPY expression (3a) and a decreased POMC expression (3b) in the arcuate nucleus. Interestingly, in conditions of transition from hunger to satiety, or acute feeding, the lack of astrocytic FoxO1 led to hyperphagia, which is coherent with increased NPY levels and increased glycemia (4). **C** Bottom right panel (from Yin et al*.,* 2024 (reference 50)): In HFD feeding conditions, increased astrocytic Tak1 function ameliorates glucose and cholesterol metabolism, via POMC neurons. This could be linked to the finding that Tak1 in hypothalamic astrocytes, via CD38 (1), mediates the transfer of mitochondria from astrocytes to POMC neurons (2), a phenomenon described to help the maintenance of cellular function or even cell survival (3). **D** Top right panel (from He et al*.,* 2024 (reference 54)): The authors have shown that chronic sleep fragmentation disrupts systemic glucose homeostasis (1) via impaired brain glucose uptake and metabolism, particularly in the hypothalamus. Elevated acetate, produced by gut microbiota (1), is associated with increased acetate levels in the circulation and the hypothalamus (2–3), and would act as a compensatory signal, enhancing hypothalamic glycolysis and TCA cycle activity through pyruvate carboxylase (4–5) activation. Central to this response are astrocytes, which regulate acetate utilization via *Acss1*, an enzyme selectively expressed in these glial cells (3). Astrocytic *Acss1* expression modulates acetate oxidation and influences insulin sensitivity and inflammatory tone (6). These findings position hypothalamic astrocytes as key mediators of acetate-driven neuro-metabolic adaptation to sleep disruption*.* Abbreviations: *Acss1*: acyl-CoA synthetase short-chain family member 1; ATP: adenosine triphosphate; CD38: cluster of differentiation 38; DMH: dorsomedial nucleus of the hypothalamus; FoxO1: forkhead box O1; GLUT1: glucose transporter 1; HFD: high-fat diet; IR: insulin receptor; NPY: neuropeptide Y; POMC: pro-opiomelanocortin; Tak1: transforming growth factor (TGF)-β-activated kinase 1; TCA: tricarboxylic acid

Over the past several years, numerous studies have investigated different pathways by which hypothalamic astrocytes actively participate in this process. GLUT1, expressed by endothelial cells at the BBB and astrocytes, plays a major role in central glucose sensing in the brain [4], and defects in GLUT1, such as those seen in genetic disorders like GLUT1 haploinsufficiency, are associated with reduced cerebral glucose uptake in humans [41]. Disruption of astrocytic glucose sensing, through targeted deletion of glucose transporters such as GLUT1 or GLUT2, or the ablation of the IR, has been shown to result in glucose intolerance, insulin resistance, and/or altered secretion of glucoregulatory hormones [4, 5, 34]. Moreover, an intracellular metabolic shift within astrocytes toward the utilization of alternative substrates, such as fatty acids, has been proposed to underlie some of these metabolic impairments [5]. However, a recent study has challenged earlier assumptions originating from previous work in mice and humans, showing that postnatal deletion of astrocytic GLUT1 can paradoxically improve both central and peripheral glucose metabolism [42]. While astrocytic GLUT1 loss in isolated astrocytes impaired glucose metabolism, its deletion in vivo enhanced brain glucose utilization and tolerance, linked with a shift toward glutamine oxidation [42]. This occurred without changes in intracellular ATP levels but was accompanied by increased ATP release in response to glucose, suggesting compensatory adaptations [42]. Elevated extracellular ATP, acting via purinergic receptors, appears central to improved glucose regulation as blocking this pathway reversed the benefits seen in the model of astrocytic GLUT1 deficiency [42]. Astrocytic GLUT1 deficiency led to the upregulation of IR in astrocytes, whereas astrocytic IR-deficiency appeared to prevent the increase in brain ATP and to lead to opposing metabolic outcomes, a phenotype which could be rescued by purinergic activation [42]. Morphologically, GLUT1 loss altered hypothalamic astrocytes, increasing the overall complexity of their processes and reducing their coverage of arcuate POMC neurons, potentially facilitating the activation of the latter [42]. Interestingly, arcuate-specific deletion of astrocytic GLUT1 was associated with improved glucose tolerance and insulin secretion, especially under HFD conditions [42]. Together, these findings suggest that astrocyte-derived ATP acts on purinergic receptors in the brain to promote metabolic homeostasis through mechanisms involving insulin signaling, and further highlight ATP as a key gliotransmitter (Fig. 1). In a complementary study, Thieren and colleagues found that GLUT1-deficient cortical astrocytes sustained normal glucose levels via enhanced glycolysis and displayed attenuated reactive hypertrophy after stroke, thereby conferring neuroprotection [43]. Despite differences in substrate preference and experimental paradigm, both studies underline the indispensable role of astrocytes in metabolic regulation and their capacity for compensatory adaptations that safeguard brain and systemic homeostasis even in the absence of a key transporter such as GLUT1.

Discrepancies between human and rodent findings regarding the effects of GLUT1 loss on brain glucose uptake may stem from cell-type-specific roles of GLUT1 in astrocytes versus endothelial cells. In humans with GLUT1 deficiency syndrome, for instance, GLUT1 is absent in both endothelial cells and astrocytes, whereas the rodent study discussed here selectively targets astrocytic GLUT1 [41, 44–46]. In addition, the dynamic nature of blood glucose regulation, the involvement of multiple GLUT isoforms, and the interplay among various glucose uptake pathways introduce further complexity into the interpretation of experimental results and the understanding of the relative contributions of different GLUTs in astrocytes.

Other studies have also explored the molecular mechanisms by which astrocytes regulate glucose metabolism. One such study targeted the role of the transcription factor Forkhead box O1 (FoxO1) in astrocytes for regulating glucose metabolism, extending prior work that focused on its function in hypothalamic neurons, where it acts downstream of insulin and leptin signaling to regulate feeding and energy balance [47, 48]. FoxO1 deletion in astrocytes did not affect basal behavior but led to increased rebound food intake and elevated blood glucose after overnight fasting followed by refeeding [49], suggesting that astrocytic FoxO1 plays a role in the acute transition from fasting to satiety. Interestingly, these metabolic changes were accompanied by increased hypothalamic neuronal activity (c-Fos immunoreactivity). More specifically, mice lacking FoxO1 in hypothalamic astrocytes showed both increased neuropeptide Y (NPY) and decreased POMC immunoreactivity in the arcuate nucleus, consistent with overactivation of orexigenic NPY neurons [49]. Furthermore, intracerebroventricular (icv) administration of the NPY antagonist BIIE0246 normalized both food intake and blood glucose levels in astrocytic FoxO1-deficient mice after refeeding, further supporting the notion that activation of hypothalamic NPY neurons mediates the acute metabolic impairments resulting from the ablation of FoxO1 in astrocytes [49]. In terms of astrocytic intracellular metabolism, unlike the deletion of GLUT1, the deletion of FoxO1 led to a general increase in mitochondrial respiration – both basal and maximal –, elevated ATP production, and reduced extracellular lactate levels, suggesting a shift toward oxidative phosphorylation (OXPHOS) and enhanced pyruvate flux into the tricarboxylic acid (TCA) cycle [49]. In response to a HFD, astrocytic FoxO1-deficient mice gained more weight, had a higher fat-to-lean mass ratio, elevated fasting blood glucose and insulin levels, and reduced insulin sensitivity [49] (Fig. 1). The study highlights astrocytic FoxO1 as a regulator of systemic glucose and energy balance, acting likely through metabolic remodeling and modulation of surrounding neuronal circuits. Although the precise mechanisms by which astrocytes influence neurons were not determined, the observed changes in ATP and lactate levels could play a role. Previous studies have shown that insulin’s ability to inhibit FoxO1 is reduced when FoxO1 is already active [47], suggesting that disruptions in other signaling pathways that regulate FoxO1, such as inflammatory cascades involving NF-κB, could in turn alter astrocyte-mediated control of neuronal activity and glucose homeostasis [47].

Moreover, Yin and colleagues sought to determine the role of transforming growth factor (TGF)-β-activated kinase 1 (Tak1) in astrocytes from the MBH, particularly in glucose homeostasis [50]. Tak1, a member of the mitogen-activated protein kinase kinase kinase (MAP3K) family, is activated by various cytokines (e.g., TGF-β and TNF-ɑ), and activates NF-κB, among others. Notably, overexpression of TGF-β1 in astrocytes has been linked to glucose intolerance [37]. Moreover, Tak1 activation increases TGF-β1 expression, suggesting a role for astrocytic Tak1 in the control of glucose homeostasis. Remarkably, exposing mice to HFD for 4 weeks led to the expected hyperglycemia and glucose intolerance, but also to increased Tak1 activity, as measured by phosphorylated Tak1 levels, specifically in the hypothalamus and primarily in MBH astrocytes, but also in microglia [50]. With a standard chow diet, mice lacking astrocytic Tak1 in the MBH displayed an impairment of glucose tolerance and insulin sensitivity [50]. Conversely, expressing a constitutively active form of Tak1 in MBH astrocytes improved these parameters, but only under HFD conditions, not with a chow diet [50]. Ablating Tak1 in astrocytes reduced glucose-induced activation of neurons across multiple hypothalamic nuclei, notably that of arcuate POMC neurons [50]. The authors further showed that Tak1’s role in glucose homeostasis relies on POMC neurons [50]. For example, chemogenetic inhibition of POMC neurons in HFD-fed mice with constitutively active Tak1 in MBH astrocytes largely abolished the previously observed beneficial effects [50]. It was later found that the ablation of Tak1 from MBH astrocytes reduced the transfer of mitochondria from astrocytes to specifically POMC neurons [50]. Mitochondria transfer is a mechanism that has been described as a beneficial process for damaged or diseased cells and would promote their survival and proper function [50]. This study further found that the role of Tak1 in mitochondrial transfer from astrocytes to POMC neurons likely depends on CD38 [50], which has been previously implicated in this process [51] and whose expression was found regulated by Tak1. Restoring CD38 expression rescued the negative effects on glucose homeostasis caused by Tak1 ablation in astrocytes [50] (Fig. 1). Interestingly, Tak1 can also regulate the expression of JNK1, a well-known inhibitor of insulin signal transduction [52, 53]. Whether astrocytic Tak1’s role in glucose homeostasis could also involve regulation of Jnk1 is yet an unexplored avenue.

He et al*.* investigated glucose homeostasis dysregulation associated with sleep disruption using a chronic sleep fragmentation (SF) mouse model [54]. SF mice exhibited impaired glucose homeostasis, including increased fasting blood glucose, reduced glucose tolerance, and lower insulin sensitivity [54]. Functional imaging of revealed decreased brain glucose uptake, particularly in the hypothalamus in SF mice when compared to controls. This occurred alongside impaired brain glycolysis, TCA cycle activity, and insulin signaling [54]. This study identified acetate metabolism, especially in hypothalamic astrocytes, as a potential compensatory mechanism. Indeed, SF mice displayed gut microbiota alterations, increased intestinal permeability, and elevated circulating and hypothalamic levels of acetate, a short-chain fatty acid primarily produced by gut bacteria [54]. Experimental evidence suggested that accumulation of acetate mitigates SF-induced metabolic impairments, by binding to and activating pyruvate carboxylase (PC), thereby enhancing glycolysis and the TCA cycle in the hypothalamus. Chronic administration of acetate, either peripherally or centrally (icv), improved glucose handling and insulin sensitivity [54]. Notably, acetate icv infusion mimicked the effects of peripheral administration and acetate was found in both cases to accumulate in the hypothalamus, reinforcing this area’s central role [54]. SF reduced acetate oxidation, its incorporation in the TCA cycle, and ATP production in the hypothalamus, likely due to decreased expression of acyl-CoA synthetase short-chain family member 1 (ACSS1), the enzyme that converts acetate to acetyl-CoA [54]. *Acss1*, only found in glial cells was predominantly expressed in astrocytes and astrocyte-specific *Acss1* ablation increased acetate levels and reversed SF-induced glucose intolerance and insulin resistance, with no effect in non-SF mice [54]. Astrocytes from the paraventricular nucleus of the hypothalamus (PVN) were the most activated ones in response to SF and reducing the expression of *Acss1* from these cells specifically improved SF-associated defects in glucose handling and insulin sensitivity [54] (Fig. 1). Conversely, *Acss1* overexpression in PVN astrocytes worsened SF-induced metabolic dysfunction [54]. Furthermore, SF mice exhibited hypothalamic inflammation, marked by increased pro-inflammatory cytokines (e.g., TNF-ɑ, IL-6) [54]. Strikingly, icv acetate administration reversed this inflammatory response, underscoring the link between hypothalamic astrocytes, metabolism, and inflammation [54]. These findings highlight acetate’s protective role in SF-induced metabolic dysfunction and further highlight astrocytes as key regulators of systemic glucose homeostasis (Fig. 1). Interestingly, previous studies have shown that peripherally administered acetate, or acetate produced by inulin fermentation in the gut leads to acetate accumulation in the hypothalamus. In this context, acetate activates arcuate neurons, but not in the ventromedial nucleus of the hypothalamus (VMH), PVN or brainstem [55]. Specifically, acetate activates arcuate POMC neurons while inhibiting AgRP neurons. On a metabolic level, acetate suppresses AMP-activated protein kinase (AMPK), leading to an activation of acetyl-CoA carboxylase (ACC). While isotopic labeling with C^13^ has demonstrated that the C^13^ from peripheral acetate is incorporated into acetate, GABA, glutamate, and glutamine in the hypothalamus [55], this does not necessarily indicate that increased glutamate levels of in the PVN mediate improvements in glucose homeostasis. In fact, chemogenetic activation of PVN astrocytes, has been associated with impaired glutamate clearance, leading to elevated extracellular glutamate which in turn enhanced excitatory neuronal firing within the PVN, increased sympathetic nervous system activity, and drove diabetic-like metabolic disturbances, including increased hepatic glucose output [7]. Taken together, these studies illustrate the role of PVN astrocytes in the regulation of systemic glucose homeostasis, whether through changes in their own cellular metabolism, or via modulation of synaptic activity, ultimately influencing peripheral organ function.

This body of research highlights the multifaceted role of astrocytes in regulating systemic glucose homeostasis, shedding light on key intracellular signaling pathways, metabolic adaptations, and astrocyte-neuron interactions that drive this process in the hypothalamus. Recent studies suggest a common mechanism in astrocyte-mediated blood glucose regulation: shifts in intracellular metabolism that favor alternative fuel utilization over glucose, thereby enhancing TCA cycle and/or oxidative phosphorylation. Additionally, various gliotransmitters have been implicated in glucoregulation, though their specificity across brain regions, microenvironments, and neuronal populations requires further investigation. A critical challenge moving forward is to unravel how metabolic and inflammatory cues interact, whether synergistically or antagonistically, in the regulation of systemic glucose homeostasis.

## Hypothalamic astrocytes: central modulators of peripheral fat metabolism and body weight regulation

Another aspect of how astrocytes regulate whole-body energy homeostasis has been the focus of some recent studies: the adipose tissue function. Hypothalamic astrocytes seem to be directly involved in the regulation of body weight, especially in conditions of hypercaloric, HFD feeding.

Two recent studies conducted by Chen and colleagues investigated the role of astrocytes located in the arcuate nucleus as key components of the central circuits regulating adipose tissue function and, by extension, body weight [56, 57].

In the first study, the activation of arcuate astrocytes, either optogenetic or chemogenetic, was found to increase both norepinephrine (NE) levels in iWAT, as revealed by the use of an NE-specific sensor and fiber photometry [56]. The activation of arcuate astrocytes was also associated with increased activating phosphorylation of the hormone-sensitive lipase (HSL), elevated glycerol levels in the iWAT, and higher circulating levels of non-esterified fatty acids, collectively suggesting enhanced lipolysis in the iWAT [56]. The authors further characterized this circuit and found that the T13-L1 paravertebral sympathetic ganglia (PG) contribute to the increased sympathetic input to iWAT induced by the activation of arcuate astrocytes [56]. Using fiber photometry, the authors finally found that arcuate astrocytes activation leads to an increase in the activity of surrounding POMC neurons. Importantly, the chemogenetic inhibition of POMC neurons, concomitantly to the activation of arcuate astrocytes, prevented the increase in NE levels in the iWAT suggesting that POMC neurons participate in the astrocytic regulation of the sympathetic outflow to the iWAT and lipolysis [56]. In summary, this study by Chen and colleagues identified a hypothalamic circuit where arcuate astrocytes activation induces the activation of POMC neurons, which mediates, via the T13-L1 PG, an increase in both the sympathetic outflow received by the iWAT and lipolysis.

In another very recent study by the same research group, Chen and colleagues have continued interrogating the role of arcuate astrocytes in the regulation of adipose tissue function, but this time, they employed a different approach which does not rely on an “astrocyte activation-based gain-of-function” [57]. By driving the expression of the diphtheria toxin receptor specifically in arcuate astrocytes via stereotactic delivery of adeno-associated viruses, followed by post-recovery intramuscular injection of diphtheria toxin, the authors did not induce a complete loss of arcuate astrocytes, but rather a loss of astrocyte processes (termed “astrocyte process loss” or “astroPL”) compared to the control group [56]. Interestingly, in line with the results of their previous study, astroPL in the arcuate nucleus was associated with increased body weight and fat/lean mass ratio, reduced lipolysis in adipose tissue (evidenced by reduced levels of activated phosphorylated HSL), increased adipocyte size, and lower energy expenditure when compared to control conditions [57]. Mice with astroPL in the arcuate nucleus under HFD-feeding conditions, when compared to their control counterparts, exhibited a higher body weight gain, as well as a higher fat/lean mass ratio [57]. This was also associated with an increase in food intake [57]. Coherently with their previous study, mice with astroPL in the arcuate nucleus displayed decreased sympathetic outflow to the iWAT, as supported by the finding of reduced levels of NE, but also the rescue of the increased body weight gain in the mice with arcuate astroPL via the chemogenetic stimulation of sympathetic terminals in the iWAT [56].

**Fig. 2:**
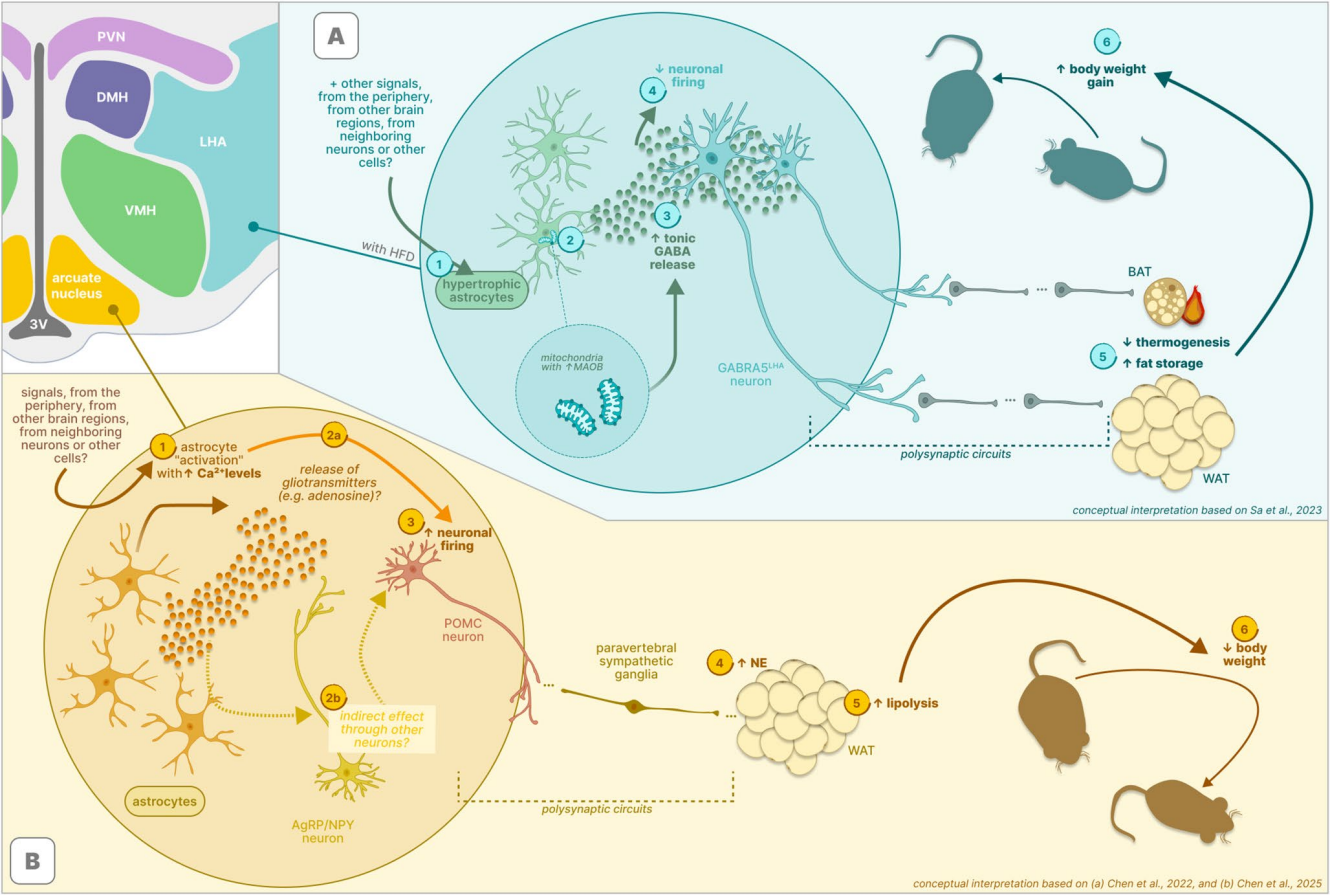
New findings reveal hypothalamic astrocytes regulate sympathetic outflow to adipose tissue and influence body weight. **A** Top panel (from Sa et al*.,* 2023) (reference 58): The authors have identified a GABAergic neuron population in the LHA (GABRA5^LHA^ neurons) which project to the BAT and WAT. In HFD-fed mice, hypertrophic LHA astrocytes (1) upregulated MAOB (2), increasing GABA production (3), and inducing tonic inhibition of GABRA5^LHA^ neurons (4). This led to reduced thermogenesis (5), increased fat storage (5), and subsequent increased body weight gain (6). Astrocyte-derived GABA appears as key to the hypothalamic control over peripheral energy storage and obesity. **B** Bottom panel (from Chen et al*.,* 2022 and 2025 (references 56, 57)): Chen et al*.* highlighted arcuate astrocytes as regulators of adipose tissue metabolism via modulation of sympathetic outflow. Activation of arcuate astrocytes (1) leads to potentially direct (2a) or indirect (2b) POMC neurons activation and, via paravertebral sympathetic ganglia, increased sympathetic outflow (4) and lipolysis (5) in iWAT. This would lead to a decrease in body weight (6). These findings identify arcuate astrocytes as central modulators of hypothalamic control over peripheral fat metabolism and body weight regulation. Abbreviations: BAT: brown adipose tissue; DMH: dorsomedial nucleus of the hypothalamus; GABA: gamma(γ)-aminobutyric acid-producing; GABRA5: α5 subunit of the GABAA receptor; HFD: high-fat diet; iWAT: inguinal white adipose tissue; LHA: lateral hypothalamic area; MAOB: monoamine oxidase B; POMC: pro-opiomelanocortin; PVN: paraventricular nucleus of the hypothalamus; VMH: ventromedial nucleus of the hypothalamus; WAT: white adipose tissue

Taken together, these two reports support a role for astrocytes in the arcuate nucleus as modulators of the activity of neighboring neurons, such as POMC neurons, whether it is directly or indirectly via modulation of other neurons and synapses, to impact downstream circuits and influence metabolic peripheral organs such as the adipose tissue, and ultimately body weight. While these studies do not fully resolve the circuits in question, the results suggest the implication of gliotransmission and the modulation of intra-hypothalamic synapses with further metabolic repercussions (Fig. 2).

In another study, Sa and colleagues sought to deepen our understanding of how the lateral hypothalamic area (LHA) contributes to the regulation of energy balance, with a particular focus on the adipose tissue [58]. They have identified an LHA neuronal population characterized by the enrichment in the α5 subunit of the GABA_A_ receptor GABRA5 (GABRA5^LHA^ neurons). These neurons project to both interscapular brown, and inguinal white adipose tissue (BAT and iWAT, respectively) [58]. This GABAergic (i.e., γ-aminobutyric acid-producing) neuronal subpopulation exhibited a decreased firing rate in mice fed a HFD. In HFD-fed mice, while the chemogenetic inhibition of the GABRA5^LHA^ neurons led to a decrease in fat thermogenesis and further weight gain, the silencing of *Gabra5* in the LHA was characterized by reduced weight gain, associated with an apparent decrease in lipid storage [58].

In the context of this review, the changes in LHA astrocytes in response to long-term HFD feeding which were uncovered by the authors are particularly relevant. First, LHA astrocytes were found hypertrophic, mostly at the borders of this nucleus, suggesting some heterogeneity, either inherent to the cells themselves, or via the signals they react to. Furthermore, mice fed a HFD,s versus a control diet, exhibited a higher gene expression of monoamine oxidase B (MAOB) in LHA astrocytes which would drive a larger production of GABA [58]. Importantly, this astrocytic GABA would mediate a tonic, i.e. extrasynaptic, inhibition of neighboring GABRA5^LHA^ neurons. Silencing the *Maob* gene in LHA astrocytes under HFD diet feeding conditions reduced body weight gain, the size of fat depots, and the size of adipocytes themselves, and increased BAT thermogenesis, without significantly affecting food intake or energy expenditure [58]. Looking into more details, the authors found that this silencing was also associated with a decrease in both the GABA tonic inhibition of LHA neurons and in the astrogliosis phenotype in the LHA [57]. A similar rescue was obtained with a treatment with KDS2010, a reversible MAOB inhibitor [58].

This study further supports the role of hypothalamic astrocytes in the (dys)regulation of body weight via regulation of the adipose tissue function. The authors speculate that a normal firing of GABRA5^LHA^ neurons, projecting to the WAT and BAT though polysynaptic circuits, normally suppress fat accumulation. Yet, in response to hypercaloric feeding, LHA astrocytes produce increased amounts of GABA which would tonically inhibit the activity of the GABRA5^LHA^ neurons, inhibiting thermogenesis, promoting fat accumulation in the WAT, and ultimately body weight gain. In this context, astrocyte-derived GABA would contribute to the pathophysiology of obesity in response to overfeeding (Fig. 2).

## Hypothalamic astrocytes: integrators of circadian, hormonal, and developmental signals in metabolic control

In recent years, some studies have uncovered new layers of complexity in the role of hypothalamic astrocytes in the control of whole-body energy metabolism and their involvement in the coordination of metabolic processes with circadian rhythms has gathered increasing interest.

The suprachiasmatic nucleus (SCN), located in the hypothalamus, is a master circadian pacemaker known to regulate daily physiological cycles, including feeding and energy expenditure, by synchronizing peripheral clocks throughout the body.

In the SCN, neurons and astrocytes cooperate to facilitate this regulation. The control of circadian rhythms relies, at least partially, on feeding cues. Conditions such as type 2 diabetes or obesity are associated with an impaired central control of whole-body energy homeostasis, and, similarly to what is observed with shift work, could be linked to impairments in circadian rhythms, such as mistimed feeding [59]. In the past recent years, several studies have investigated the role of hypothalamic astrocytes, in and out of the SCN, in integrating and sending cues to regulate energy metabolism according to circadian rhythms, and the implication of such function for whole-body metabolic disturbances.

**Fig. 3:**
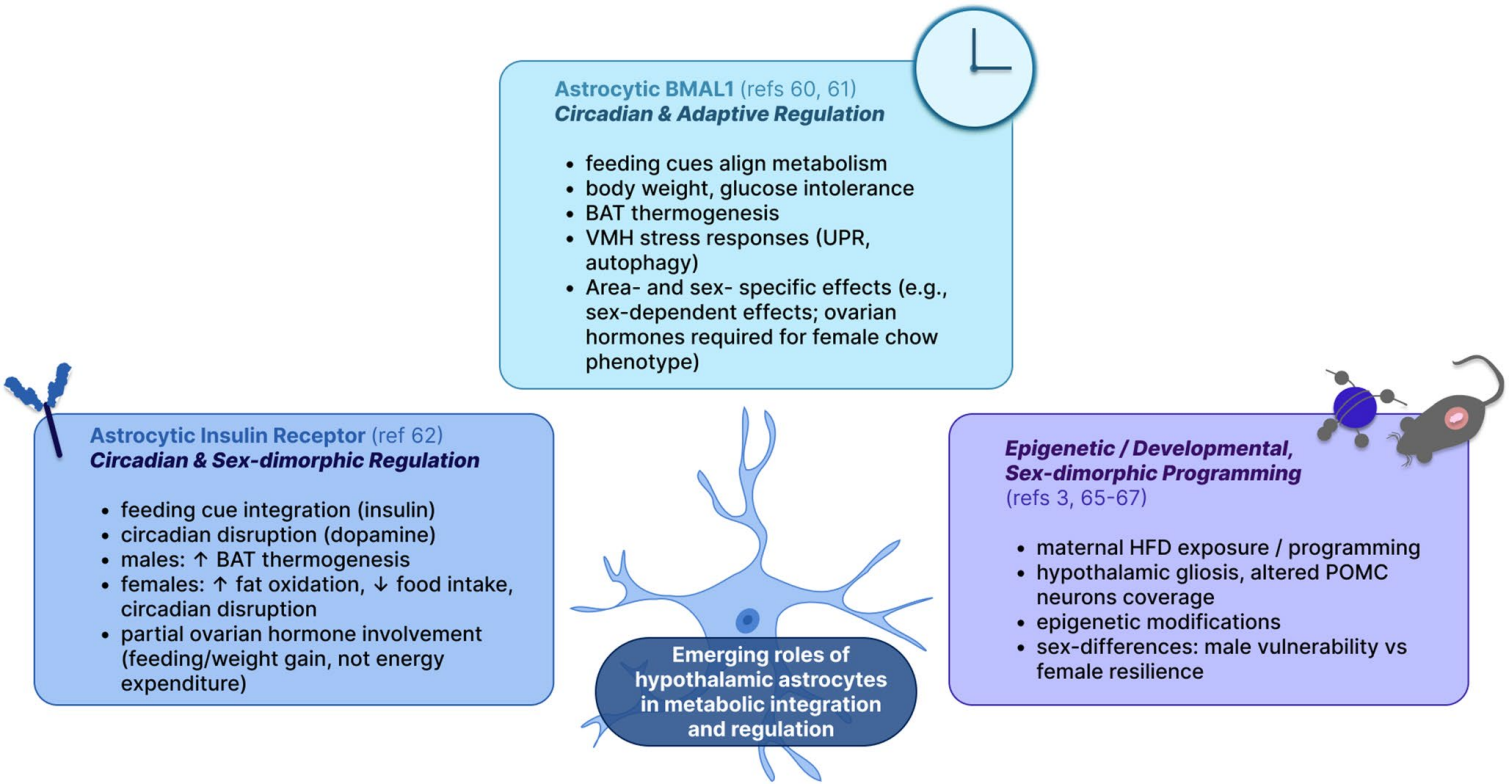
Emerging roles of hypothalamic astrocytes in integrating circadian, hormonal, and developmental signals to shape systemic energy balance. Astrocytic BMAL1 links feeding cues to body weight, glucose tolerance, and BAT thermogenesis, with loss of BMAL1 triggering VMH stress responses (UPR, autophagy) and revealing sex- and ovarian hormone-dependent effects. Astrocytic IR function as metabolic-circadian integrator; its deletion disrupts feeding cue responses and circadian rhythms via dopamine signaling, producing sex-divergent outcomes, partly independent of ovarian hormones. Epigenetic and developmental programming, notably through maternal high-fat diet exposure, induces astrocytic gliosis, altered neuronal coverage, and epigenetic modifications, conferring male vulnerability versus female resilience. Together, these findings position astrocytes as central integrators of emerging regulatory layers in energy metabolism. Abbreviations: BMAL1: basic helix-loop-helix ARNT like 1; BAT: brown adipose tissue; HFD: high-fat diet; IR: insulin receptor; POMC: pro-opiomelanocortin; UPR: unfolded protein response; VMH: ventromedial hypothalamus

For example, studies disrupting the core clock gene basic helix-loop-helix ARNT like 1 (*Bmal1)* specifically in astrocytes have uncovered notable effects on body weight, glucose homeostasis, and energy expenditure. One study demonstrated that global astrocytic deletion of *Bmal1* prevented the development of DIO in mice without altering food intake or locomotion, but rather by increasing BAT thermogenic activity [60]. Furthermore, the lack of *Bmal1* in astrocytes led to improved glucose tolerance in HFD conditions as well, possibly via an improved sensibility of glucose-responsive neurons in the dorsomedial region of the VMH [60]. Transcriptomic characterization of these mice suggests that the induction of cellular stress responses in the VMH (e.g., unfolded protein response in the endoplasmic reticulum, autophagy) which would lead to an increase in BAT activity and function, could mediate the phenotype [60]. Overall, these results suggest a role for astrocyte-derived stress signaling in driving peripheral metabolic adaptations (Fig. 3).

A study by Luengo-Mateos and colleagues further explored the role of astrocytic BMAL1 in hypothalamic regulation of energy metabolism [61]. In male mice, conditional deletion of *Bmal1* in all astrocytes led to increased body weight when animals were maintained on a standard diet [61], an outcome notably opposite to that observed in the study previously discussed where astrocytic *Bmal1* deletion was constitutive [60]. In contrast, virus-mediated knock down of *Bmal1* specifically in hypothalamic astrocytes did not induce any significant change in body weight while it resulted in increased food intake and improved glucose tolerance [61], suggesting a dissociation between energy intake and body weight outcomes, and that local astrocytic clocks can influence specific aspects of metabolic control. Further than highlighting different phenotypes depending on the model used and a putatively area-specific role of the astrocytic clock in the regulation of energy metabolism, this study also identified pronounced sex-dependent effects. In female mice, both global and MBH-specific astrocytic *Bmal1* deletion led to increased body weight despite increased energy expenditure and elevated BAT thermogenesis on a chow diet [61]. Under HFD conditions, however, these same models displayed a male-like obese phenotype, characterized by increased food intake, glucose intolerance, and reduced energy expenditure [61]. Ovariectomy reversed the chow diet-associated weight gain, suggesting a role of ovarian hormones in the observed sex-specific responses [61]. In addition, deleting *Bmal1* from arcuate astrocytes specifically did not affect body weight, body composition, or glucose tolerance, but was associated with reduced food intake, further supporting that astrocytes operate in a region-specific manner in regard to their role in the regulation of circadian function [61]. Overall, these findings underscore the complexity of astrocytic BMAL1 function in energy metabolism, with effects that depend not only on the spatial and temporal context of gene disruption but also on sex and hormonal status (Fig. 3).

Astrocytes have been shown to play a crucial role in regulating energy metabolism, particularly through nutrient and hormone sensing, including insulin [5]. Building on this understanding, a recent study led by González-Vila explored the specific role of astrocytic insulin signaling in influencing circadian rhythms and energy homeostasis [62]. The authors found that astrocytic IR plays a role as metabolic-circadian integrator, in a sex-dimorphic manner. Females with conditional ablation of astrocytic IR displayed impaired light-entrained and free-running locomotor activity, as well as reduced amplitude of activity rhythms, independent of ovarian hormones [62]. Males showed a lack of flexibility regarding their food anticipatory activity in response to insulin under restricted feeding schedule [62]. These circadian alterations associated with compromised astrocytic IR function seemed to implicate changes in dopamine signaling via the DRD2. In the hypothalamus specifically, catecholamine synthesis was upregulated and associated with increased dopamine and metabolites levels [62]. Metabolically, loss of astrocytic IR led to reduced body weight, fat, and lean mass under standard diet conditions [62]. The deletion of IR in astrocytes was associated with increased energy expenditure in both sexes but was linked to reduced food intake and a shift toward fat oxidation only in females [62]. Circulating metabolic factors were differentially affected in males and females: in males, levels of leptin, triglycerides, and cholesterol were reduced, whereas in females, triglycerides were elevated alongside improved glucose tolerance [62]. Ovarian hormones were found to be involved in the feeding and weight gain phenotype observed in females while energy expenditure remained elevated, which indicated the existence of both estrogen-dependent and -independent effects of astrocytic IR [61]. Under HFD conditions, both males and females lacking astrocytic IR were resistant to body weight gain [62]. In males, this seemed primarily linked to increased BAT thermogenesis without changes in food intake [62]. In contrast, females showed a combined phenotype of increased energy expenditure, reduced food intake, and heightened physical activity [62]. Notably, the shift toward fat oxidation observed in females under standard diet was maintained under HFD [62]. Collectively, these findings suggest that astrocytic insulin signaling promotes energy storage and adiposity. Astrocytic IR may coordinate feeding cues with systemic metabolic responses, while its disruption appears to lead to sex-divergent adaptations that confer protection against obesity at the potential cost of circadian disruption.

Although these studies do not fully elucidate the mechanisms by which astrocytes contribute to circadian rhythms or the central regulation of energy metabolism, they point toward a potential involvement of dopamine signaling, and several studies also suggest, at least in the SCN, that astrocytes control the extracellular levels of GABA in a circadian manner to control network timekeeping via modulation of GABAergic inhibition of neurons in the SCN [63, 64].

Epigenetic programming represents an increasingly important dimension in understanding astrocyte-mediated regulation of energy metabolism. Environmental changes during critical developmental windows, such as those occurring pre- and during pregnancy, can profoundly shape offspring metabolic health through long-lasting epigenetic modifications [65, 66]. Elgazzaz and colleagues recently investigated how maternal consumption of a hypercaloric diet (HCD), also described as Western diet (rich in fat and sugar), affects neuronal plasticity and cardiometabolic function in offspring [66]. In their study, dams were fed an HCD before and during pregnancy and either continued on HCD through lactation (“programmed”) or switched to a regular diet (RD, “non-programmed”). Offspring were then maintained on either HCD or RD for three months post-weaning. The results revealed that male offspring exposed to maternal HCD throughout development exhibited notable cardiometabolic disturbances, including elevated sympathetic tone, hypertension, and impaired glucose tolerance, even when fed a regular diet after weaning [67]. Interestingly, when these “programmed” males remained on HCD into adulthood, they showed improvements in insulin sensitivity, glucose handling, and leptin levels, suggesting an adaptive metabolic reprogramming effect [67]. Remarkably, these outcomes were not observed in female offspring, who were largely protected from such impairments despite identical maternal exposures [67]. This male-specific vulnerability was associated with hypothalamic gliosis, particularly in the PVN, alongside epigenetic modifications (gene methylation levels and miRNA expression) in genes linked to glial and astrocyte differentiation, inflammatory pathways, and neuronal plasticity [67]. Further analyses revealed that male offspring specifically exhibited blunted activation of key inflammatory pathways, including NF-κB and TGFβ, in response to postnatal HCD [67]. These findings suggest that male hypothalamic circuits may be more susceptible to early-life nutritional insults, potentially due to differences in hormonal milieu, neurodevelopmental timing, or glial reactivity (Fig. 3).

Supporting these observations, maternal HFD consumption was shown to lead to structural and functional alterations in hypothalamic astrocytes of offspring, including increased glial coverage of POMC neurons, modified expression of glutamate and glucose transporters, and disrupted glucose sensing [3]. Overall, results suggest that astrocytes could not only act as key regulators of metabolic adaptation in the adult life but may also be central players in mediating the long-term impact of maternal diet on hypothalamic function.

Overall, these findings underscore an evolving view of hypothalamic astrocytes as active, integrative regulators of systemic metabolism. Linking circadian timing, hormonal cues, and early-life nutritional exposures, astrocytes could play both immediate and long-term roles in shaping energy balance through mechanisms that are in addition highly region- and sex-specific. Their involvement in overall epigenetic programming further highlights their potential as mediators of metabolic health across the lifespan.

## Concluding remarks and future perspectives

Over the past few years, our understanding of astrocytes, particularly those residing in the hypothalamus, and their involvement in the central regulation of energy metabolism has made considerable progress. Growing evidence has shifted the field from a neuron-centric view toward a more integrated perspective that recognizes astrocytes as essential partners to neurons. These glial cells engage in complex, dynamic interactions with neurons that are fundamental for nutrient and hormone sensing, gliotransmission, and the regulation of neural circuits controlling both central and peripheral processes, including sympathetic nervous system activity.

What distinguishes hypothalamic astrocytes from those in other brain regions remains an open question. One plausible explanation lies in their unique anatomical and cellular environment. Hypothalamic astrocytes are strategically positioned in close association with the vasculature and are particularly enriched in regions where the BBB is more permeable, such as the areas adjacent to the ME and the 3 V. In these regions, they also interact closely with specialized glial cells known as tanycytes, which are uniquely enriched in the hypothalamus and contribute to barrier regulation, nutrient sensing, and signal relay between the cerebrospinal fluid and the brain parenchyma. Another distinctive feature of the hypothalamus is the presence of melanocortin neurons, such as POMC and AgRP neurons, which are molecularly specialized to rapidly respond to changes in nutrient and hormone availability. The high metabolic demands and fast responsiveness of these neurons may require close functional and synaptic coupling with adjacent astrocytes, whose dynamic support ensures proper neuronal activity. To meet these demands, hypothalamic astrocytes can transiently alter their physiological state and molecular profile, a plasticity that is particularly relevant in the regulation of energy balance and appears especially vulnerable during the early stages of DIO. In fact, recent studies have further highlighted their involvement in regulating glucose and lipid metabolism, not only through metabolic shifts within the astrocytes themselves but also via modulation of arcuate POMC and AgRP neurons. These interactions contribute to critical physiological processes including glucose counterregulation, lipolysis, thermogenesis, and feeding behavior. This combination of anatomical accessibility and unique cellular partners such as tanycytes, and neuron types with specialized metabolic functions creates a distinct glial niche, setting hypothalamic astrocytes apart from those in other brain areas and enabling them to effectively detect and integrate peripheral metabolic cues.

In addition to their metabolic functions, astrocytes are emerging as key integrators of temporal and contextual information. They appear to modulate circadian and metabolic rhythms within hypothalamic circuits, helping align physiological outputs with behavioral states and environmental cues such as light–dark cycles and feeding schedules. The influence of sex as a biological variable and developmental history is also becoming increasingly apparent. Astrocytic responses are shaped by nutrient/hormonal status, as well as early-life nutritional exposures which may program long-term glial function through epigenetic mechanisms. These findings add yet another layer of complexity to our understanding of astrocytic plasticity and underscore the need for further investigation.

Technological advances have greatly enriched our understanding of the diversity, adaptability, plasticity, and function of hypothalamic astrocytes. However, dissecting the specific roles of astrocytes within distinct hypothalamic nuclei remains technically challenging. Most currently available viral tools rely on pan-astrocytic promoters, which do not allow for selective targeting of astrocytes in specific nuclei or neural circuits. Overcoming this limitation will require the identification of astrocyte subpopulation markers, if they exist, that enable precise, region-specific manipulation. Ideally, such markers could also enable systemic (e.g., intravenous) delivery of genetic tools, avoiding the need for invasive stereotaxic injections that can cause local brain injury. An alternative and complementary strategy may involve targeting astrocytes based on the neuronal populations they interact with, providing an indirect yet potentially powerful means to study circuit-specific astrocytic functions. Such approaches may yield valuable insights into the molecular mechanisms by which astrocytes regulate metabolism, including their interactions with defined neuronal subtypes, and how these processes are altered in disease states such as obesity and type 2 diabetes.

In conclusion, hypothalamic astrocytes are versatile and responsive integrators of systemic and environmental signals. As central players in the brain (hypothalamus)-body axis, they represent a promising focus for understanding and treating metabolic disorders. By continuing to explore (astro)glia-neuron partnerships as well as other critical compounds that shape the cell–cell interaction such as extracellular matrix, or interactions with other glial populations such as microglia, rather than focusing solely on neuronal mechanisms, we may uncover novel therapeutic strategies to restore or enhance energy homeostasis in obesity and related conditions.

## Data Availability

No datasets were generated or analysed during the current study.
